# Effect of Lipopolysaccharide and TNF*α* on Neuronal Ascorbic Acid Uptake

**DOI:** 10.1155/2021/4157132

**Published:** 2021-07-03

**Authors:** Veedamali S. Subramanian, Trevor Teafatiller, Anshu Agrawal, Masashi Kitazawa, Jonathan S. Marchant

**Affiliations:** ^1^Department of Medicine, University of California, Irvine, CA 92697, USA; ^2^Department of Environmental and Occupational Health, University of California, Irvine, CA 92697, USA; ^3^Department of Cell Biology, Neurobiology, and Anatomy, Medical College of Wisconsin, Milwaukee, WI 53226, USA

## Abstract

Vitamin C (ascorbic acid: AA) uptake in neurons occurs via the sodium-dependent vitamin C transporter-2 (SVCT2), which is highly expressed in the central nervous system (CNS). During chronic neuroinflammation or infection, CNS levels of lipopolysaccharide (LPS) and LPS-induced tumor necrosis factor-*α* (TNF*α*) are increased. Elevated levels of LPS and TNF*α* have been associated with neurodegenerative diseases together with reduced levels of AA. However, little is known about the impacts of LPS and TNF*α* on neuronal AA uptake. The objective of this study was to examine the effect of LPS and TNF*α* on SVCT2 expression and function using *in vitro* and *in vivo* approaches. Treatment of SH-SY5Y cells with either LPS or TNF*α* inhibited AA uptake. This reduced uptake was associated with a significant decrease in SVCT2 protein and mRNA levels. *In vivo* exposure to LPS or TNF*α* also decreased SVCT2 protein and mRNA levels in mouse brains. Both LPS and TNF*α* decreased *SLC23A2* promoter activity. Further, the inhibitory effect of LPS on a minimal *SLC23A2* promoter was attenuated when either the binding site for the transcription factor Sp1 was mutated or cells were treated with the NF-*κ*B inhibitor, celastrol. We conclude that inflammatory signals suppress AA uptake by impairing *SLC23A2* transcription through opposing regulation of Sp1 and NF-*κ*B factors.

## 1. Introduction

Vitamin C (ascorbic acid: AA) is an essential micronutrient for cellular function, growth, and development, serving as a cofactor for an array of biological reactions and as a pleiotropic intracellular antioxidant [1, 2]. AA also serves as a first-line antioxidant defense to neutralize reactive oxygen species (ROS) by promoting the regeneration of endogenous antioxidants [3]. Brain tissue is susceptible to free radical damage and oxidative stress, since the brain is the most metabolically active organ in the body, and for this reason, the brain contains the highest concentration of vitamin C [3]. Accumulation of vitamin C in the brain cells occurs by a two-step mechanism, first by absorption across the choroid plexus and second by concentration into neurons and glia [4, 5]. The human sodium-dependent vitamin C transporter-2 (hSVCT2, the product of the *SLC23A2* gene) controls these steps [4, 5]; knockout of murine SVCT2 results in undetectable levels of AA in the mouse brain [6].

Deficiencies of vitamin C could play a major role in brain dysfunction and neurodegeneration. Plasma vitamin C levels are found to be significantly lower in patients with neurodegenerative diseases [3, 7–10]. For instance, in Alzheimer's disease (AD), reduced vitamin C levels may accelerate amyloid-beta (A*β*) accumulation and cognitive impairment [3, 7, 8, 11]. Reciprocally, restoration of vitamin C levels and maintaining its homeostasis appear to safeguard against cognitive decline and the progression of AD neuropathology [12]. Therefore, studies aimed at understanding the underlying molecular mechanisms that control vitamin C homeostasis in the CNS may prove essential for developing strategies to counteract conditions of disease-enhanced oxidative stress through optimization of vitamin C homeostasis.

Neuroinflammation plays a pivotal role in the pathophysiology of many neurodegenerative diseases [13–16]. Chronic neuroinflammation and systemic bacterial infection lead to increased levels of proinflammatory cytokines like TNF*α*, IL-6, and IL-1*β* [17–20]. Lipopolysaccharide (LPS) is a cell wall-derived endotoxin of most gram-negative bacteria that is capable of inducing a strong neuroinflammatory response [21, 22]. Recent studies have shown that LPS and bacterial components are associated with plaques in postmortem AD brains [21, 23]. In addition, LPS is present in a septic patient's blood plasma [24–26], where it is assumed to play an important role in systemic inflammatory response syndrome, and also, some evidence indicates that sepsis is associated with lower blood vitamin C levels [27–29]. LPS is known to affect the function and expression of certain neuronal transporters [30–32]; however, the effect of LPS and subsequently induced TNF*α* on AA uptake and SVCT2 expression has been overlooked. Therefore, we studied the impact of LPS and TNF*α* exposure on neuronal AA uptake using both *in vitro* (SH-SY5Y cells) and *in vivo* (mouse) models.

## 2. Materials and Methods

### 2.1. Materials

The ^14^C-AA (specific activity 2.8 mCi/mmol; radiochemical purity > 98%) used in vitamin uptake analysis was acquired from PerkinElmer, Inc. (Boston, MA). LPS (*E. coli* 0111:B4) was purchased from Millipore Sigma (St. Louis, MO). Human TNF*α* was bought from Invitrogen (Carlsbad, CA), and murine TNF*α* was from PeproTech, Inc. (Rocky Hill, NJ). Antibodies were obtained from the following sources: anti-*β*-actin antibodies (ThermoFisher Scientific, Huntington Beach, CA), anti-NF-*κ*B p65 and anti-IKK*αβ* antibodies (Abcam, Cambridge, MA), and anti-laminin antibodies (Santa Cruz Biotechnology, Inc., Santa Cruz, CA). LI-COR (Lincoln, NE) IRDyes 800CW and 680LT goat anti-mouse and anti-rabbit secondary antibodies were used for western blot. Celastrol was ordered from InvivoGen, Inc. (San Diego, CA). Services provided by Integrated DNA Technologies (San Diego, CA) were used to synthesize oligonucleotide primers (Table 1). All other molecular biology-grade chemicals, reagents, and materials used in this study were from commercial sources.

### 2.2. Culturing of SH-SY5Y Cells and AA Uptake Analysis

Human-derived neuroblastoma SH-SY5Y cells (ATCC, Manassas, VA) were given DMEM-F12 medium (ATCC) (with 20% fetal bovine serum (FBS) and penicillin-streptomycin added) and cultured in a temperature-controlled CO_2_ incubator at 37°C. In assays treating SH-SY5Y cells with LPS or TNF*α*, overnight serum starvation was followed by exposure either with LPS (20 *μ*g/ml) or TNF*α* (20 ng/ml) or LPS plus celastrol (100 nM) in DMEM-F12 (with 0.5% FBS and no antibiotics added). SH-SY5Y cells were subjected to pretreatment with celastrol (100 nM) for 5 h before LPS treatment. After 48 h of LPS or TNF*α* or LPS plus celastrol treatment, ^14^C-AA uptake was performed *in vitro* [33, 34]. Briefly, SH-SY5Y cells were incubated (30 min) with ^14^C-AA (0.1 *μ*Ci) in Krebs-Ringer (KR) buffer at 37°C in a water bath, and then, sample lysates were prepared for radioactivity determination using a liquid scintillation counter [33, 34].

### 2.3. Animal Studies

For *in vivo* experiments, adult male C57BL/6 mice aged 8-12 weeks (Jackson Laboratory, Bar Harbor, ME) were administered either a single injection of LPS (5 mg/kg body weight; 100 *μ*l of PBS) [33, 35] or TNF*α* (15 *μ*g/mouse; 100 *μ*l of PBS) [34, 36] or vehicle alone (100 *μ*l of PBS) intraperitoneally (IP). After 72 h, mouse brains were removed immediately after euthanization and protein and RNA extracted. The animal protocol gained approval from the Institutional Animal Care and Use Committee (IACUC), Veteran Administration Medical Center, Long Beach, CA, and University of California, Irvine, CA.

### 2.4. Real-Time PCR (RT-qPCR) Analysis

Total RNA was prepared from SH-SY5Y cells and mouse brain exposed to either LPS or TNF*α* or LPS plus celastrol and their respective controls using the TRIzol reagent (Life Technologies). One microgram of total RNA sample was reverse-transcribed (RT) to cDNA using the i-Script cDNA synthesis kit (Bio-Rad, CA). RT-qPCR analysis was then performed using the cDNA and gene-specific primers (Table 1) in a CFX96 Real-Time iCycler (Bio-Rad) [33, 34]. Simultaneously amplified *β*-actin acted as a base for comparison to normalize the relative expression of different mRNAs, which were quantified using a relative relationship method (Bio-Rad) [33, 34].

### 2.5. Heterogeneous Nuclear RNA (hnRNA) Analysis

Table 1 shows the gene-specific hnRNA primers for *Slc23a2* that were utilized in RT-qPCR analysis of total RNA prepared using LPS- or TNF*α*-treated mouse brain samples and parallel controls [37]. DNase I (Invitrogen) was added to the RNA samples for digestion before they were reverse-transcribed using i-Script cDNA synthesis kit reagents (Bio-Rad). *β*-Actin was again used to normalize RT-qPCR data, which was calculated as described above.

### 2.6. Transfection and Promoter Assays

SH-SY5Y cells were cultured on twelve-well plates (Corning) and cotransfected using Lipofectamine 2000 (3 *μ*l/well; Invitrogen) with full-length, minimal, or mutant *SLC23A2* promoter constructs (3 *μ*g plasmid DNA/well) [38, 39] and *Renilla* luciferase-thymidine kinase (pRL-TK, 100 ng/well; Promega). Cells were left to incubate for 24 h before being treated with either LPS or TNF*α* or LPS plus celastrol for an additional 48 h. Then, the samples were processed following the Promega Dual-Luciferase Reporter Assay System. In short, each sample was lysed using passive lysis buffer (Promega), and a luminometer detected both the *firefly* and *Renilla* luciferase activities sequentially [38, 39].

### 2.7. Western Blotting

SH-SY5Y cells and mouse brain total protein were prepared by homogenization in RIPA (Radioimmunoprecipitation Assay) Buffer (Sigma) with 1X protease inhibitor cocktail (Roche, Nutley, NJ). The nuclear and cytosolic fractions from LPS- or LPS plus celastrol-treated SH-SY5Y cells were obtained using the NE-PER nuclear and cytoplasmic extraction kit (ThermoFisher Scientific). Total protein (60 *μ*g) was separated using 4-12% NuPAGE Bis-Tris protein gels (Invitrogen) and transferred to a PVDF membrane. After protein transfer, the membrane was blocked for 10 min at room temperature in LI-COR Odyssey Blocking Buffer and then probed with previously characterized primary SVCT2 antibodies (1 : 500 dilution) [40], anti-IKK*αβ* antibodies (1 : 1000 dilution; Abcam), anti-NF-*κβ* p65 antibodies (1 : 1000 dilution; Abcam), anti-laminin antibodies (1 : 300 dilution; Santa Cruz Biotechnology), and anti-*β*-actin mouse monoclonal antibody (1 : 3000 dilution; ThermoFisher Scientific) used. The respective secondary antibodies (anti-rabbit IRDye-800 and anti-mouse IRDye-680, LI-COR Biosciences) were used in 1 : 30,000 dilutions [33, 34, 40]. Odyssey Infrared imaging system (LI-COR Biosciences) software was used to quantify the densitometry of specific band signal intensities normalized against *β*-actin.

### 2.8. Statistical Analyses

Carrier-mediated AA uptake analysis data from these investigations are presented as the means ± SE of at least 3 to 4 separate investigations with multiple determinations and represent a percentage relative to simultaneously performed untreated controls. RT-qPCR, western blot, and promoter assays were determined from at least 3 different batches of cells or 3 pairs of mouse samples. Student's *t*-test with *P* < 0.05 set as statistically significant was chosen to perform statistical analysis.

## 3. Results

### 3.1. Effect of LPS on hSVCT2 Function *In Vitro*

**Figure 1:**
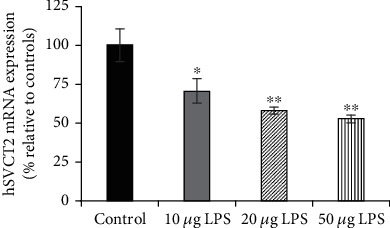
Effect of different concentrations of LPS on hSVCT2 mRNA expression in SH-SY5Y cells. SH-SY5Y cells were serum-deprived overnight and exposed to different concentrations of LPS (10, 20, and 50 *μ*g/ml). After 48 h, total RNA was prepared to carry out RT-qPCR. Data are means ± SE of at least 6 separate determinations utilizing multiple batches of SH-SY5Y cells. ^∗∗^*P* < 0.01; ^∗^*P* < 0.05.

Recent studies have shown detectable levels of LPS in the AD brain [21, 23]. LPS is a potent inflammatory stimulator, which can affect the neuronal transport of many different substrates [30–32]. To assess the effect of LPS on AA uptake, we measured hSVCT2 mRNA levels by RT-qPCR after exposure of cells to various concentrations of LPS (10-50 *μ*g/ml for 48 h). Data showed a concentration-dependent decrease in hSVCT2 mRNA expression relative to untreated control SH-SY5Y cells (Figure 1). To address the specificity of the LPS effect, we also determined mRNA levels of the brain-specific human riboflavin transporter-2 (hRFVT2) [41]. There was no significant change in hRFVT2 mRNA in SH-SY5Y cells treated with LPS (20 *μ*g) compared to untreated cells (100 ± 11 and 117 ± 20 for control and LPS treatment, respectively).

**Figure 2:**
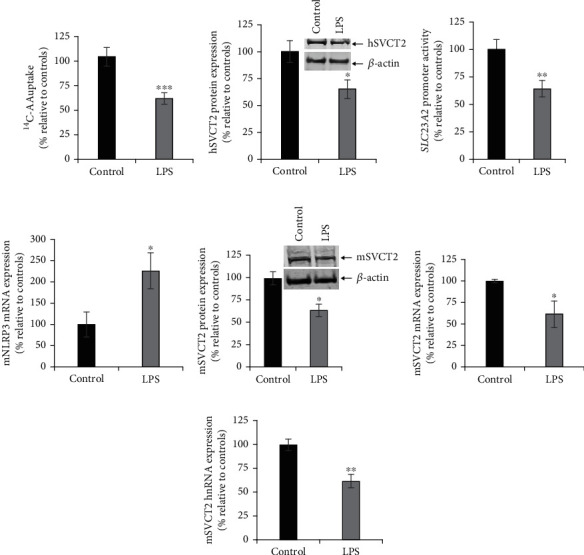
Effect of exposure of SH-SY5Y cells and mouse brain with LPS on different aspects of SVCT2 functional expression. (a) SH-SY5Y cells were serum-deprived overnight and exposed to LPS (20 *μ*g/ml). After 48 h, AA uptake was determined. (b) SH-SY5Y cells were exposed to LPS for 48 h, and the protein was prepared to perform western blot analysis to determine the hSVCT2 protein expression levels. (c) *SLC23A2* full-length promoter activity was determined in LPS-treated SH-SY5Y cells. (d, f) Total RNA isolated from LPS-administered and control mouse brain were used to determine the mSVCT2 and NLRP3 mRNA expression levels by RT-qPCR. (e) Protein samples prepared from mouse brain tissue of LPS (5 mg/kg body weight; 72 h) exposed and controls were subjected to western blotting to determine mSVCT2 protein expression levels. (g) Total RNA prepared from LPS-injected and control mouse brain were subjected to RT-qPCR to determine the mSVCT2 hnRNA expression levels. Values are means ± SE of at least 3-5 independent investigations utilizing multiple batches of SH-SY5Y cells or at least 3-5 pairs of mice. ^∗∗∗^*P* < 0.001; ^∗∗^*P* < 0.01; ^∗^*P* < 0.05.

An LPS treatment paradigm of 20 *μ*g/ml for 48 h also caused a significant (*P* < 0.001) inhibition of AA uptake (Figure 2(a)), coupled with a significant (*P* < 0.05) decrease in hSVCT2 protein expression (Figure 2(b)). The action of LPS was then interrogated at the level of the *SLC23A2* promoter, by monitoring promoter activity of a luciferase reporter construct (pGL3-*SLC23A2*). This reporter construct was transiently transfected into SH-SY5Y cells, and then, cells were treated (24 h after transfection) with LPS (20 *μ*g/ml for 48 h) before *firefly* luciferase activity was determined. LPS treatment caused significantly (*P* < 0.01) reduced *SLC23A2* promoter activity when compared to untreated SH-SY5Y cells (Figure 2(c)). These data suggest that LPS decreases hSVCT2 function via transcriptional regulation.

### 3.2. Effect of LPS on SVCT2 Function *In Vivo*

Next, we examined whether similar effects occurred *in vivo*. LPS (5 mg/kg body weight; single dose [33, 35]) was administered intraperitoneally to wild-type (WT) mice, and responses were compared with vehicle (PBS)-injected controls. To monitor inflammation, the expression of nucleotide-binding, oligomerization domain- (NOD-) like receptor family, pyrin domain containing 3 (NLRP3) was examined. NLRP3 mRNA levels were found to be significantly (*P* < 0.05) increased in LPS-administered brain samples 72 h after injection (Figure 2(d)). These data demonstrate activation of an inflammatory marker following LPS administration in mouse brain samples. As expected, TNF*α* mRNA expression was also significantly (*P* < 0.0001) increased in LPS-administered mouse brain compared to controls (100 ± 19 and 351 ± 33 for control and LPS-administered mouse brains, respectively). Levels of mSVCT2 protein, mRNA, and heterogeneous nuclear RNA (hnRNA) were then determined in control and LPS-injected animals. Results showed that the expression levels of mSVCT2 protein, mRNA, and hnRNA were all markedly reduced in LPS-injected mouse brain samples versus controls (Figures 2(e)–2(g)). The latter represents the initial products of gene transcription, reflecting the rate of transcription of a given gene [37]. Collectively, these findings suggest that LPS also decreases the mSVCT2 functional expression *in vivo*, and this occurs via a transcriptional mechanism.

### 3.3. Effect of TNF*α* on SVCT2 Function

**Figure 3:**
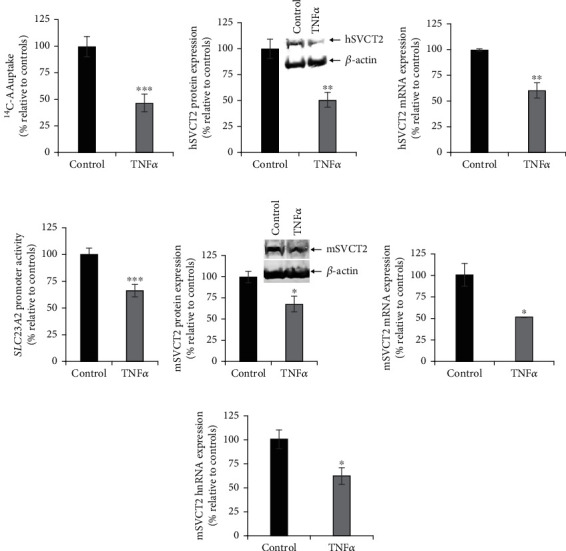
Effect of exposure of SH-SY5Y cells and mouse brain with TNF*α* on different aspects of SVCT2 functional expression. (a) SH-SY5Y cells were serum-deprived overnight and exposed to TNF*α* (20 ng/ml). After 48 h, AA uptake was performed. (b) SH-SY5Y cells were pretreated with TNF*α* for 48 h, and the total protein was prepared and used to perform western blotting to determine hSVCT2 protein expression levels. (c) Total RNA isolated from TNF*α* pretreated SH-SY5Y cells was utilized to determine the level of hSVCT2 mRNA expression by RT-qPCR. (d) *SLC23A2* full-length promoter activity was determined in TNF*α* pretreated SH-SY5Y cells. (e) Protein samples from mouse brain tissue of TNF*α* (15 *μ*g/mouse; 72 h) exposed and control mice were isolated to perform western analysis to determine mSVCT2 protein expression levels. (f) Total RNA isolated from TNF*α*-injected and control mouse brain was used to determine the mSVCT2 mRNA expression by RT-qPCR. (g) Total RNA samples prepared from TNF*α*-administered and control mouse brain were subjected to determination of the mSVCT2 hnRNA expression levels by RT-qPCR. Values are means ± SE of at least 3-5 separate investigations using different batches of SH-SY5Y cells or at least 3-4 pairs of mice. ^∗∗∗^*P* < 0.001; ^∗∗^*P* < 0.01; ^∗^*P* < 0.05.

Elevated levels of proinflammatory cytokines such as TNF*α*, IL-6, and IL-1*β* in the brain and blood are linked to neuroinflammation and systemic bacterial infection [17–20]. TNF*α* is upregulated in AD brain samples and in the blood of patients infected with bacteria [18, 20]. Still, there is little evidence to describe the effect of TNF*α* on SVCT2 expression and function in neuronal systems. Treatment of SH-SY5Y cells with TNF*α* (20 ng/ml) significantly (*P* < 0.001) inhibited AA uptake (Figure 3(a)). This inhibition in uptake was again accompanied by marked decreases in the hSVCT2 protein (Figure 3(b)) and mRNA (Figure 3(c)) expression levels, as well as a significant reduction in *SLC23A2* promoter activity (Figure 3(d)). To assess responses to TNF*α in vivo*, mice were injected intraperitoneally with TNF*α* (15 *μ*g/mouse) [34, 36], followed by evaluation of mSVCT2 protein, mRNA, and hnRNA expression levels in mouse brain after 72 h. Results showed a significant (*P* < 0.05 for all) decrease in mSVCT2 protein, mRNA, and hnRNA in TNF*α*-administrated mouse brain samples compared to control mouse brain samples (Figures 3(e)–3(g)). Together, these results suggest that the TNF*α*-mediated decrease in SVCT2 functional expression also occurs via transcriptional mechanism(s).

### 3.4. Role of the Transcription Factor Sp1 in the Effect of LPS on Neuronal AA Uptake

**Figure 4:**
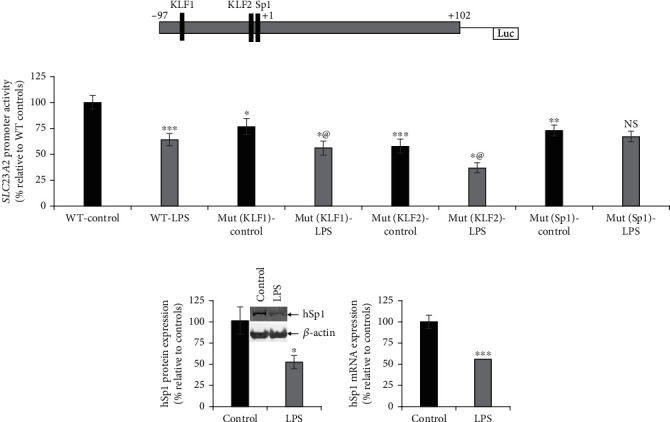
Effect of pretreating SH-SY5Y cells with LPS on the activity of *SLC23A2* minimal (WT) and mutant promoter constructs. (a) Schematic depiction of *SLC23A2* minimal promoter and the locations of Sp1- and KLF-binding sites. (b) *SLC23A2* minimal (WT), Sp1- and KLF-binding sites mutated promoter construct activities were determined in LPS-exposed and control SH-SY5Y cells (KLF1 designates the site 5′ of the second site named KLF2). Western blot analysis was done to determine the expression levels of Sp1 protein (c), and RT-qPCR was done to determine the level of Sp1 mRNA (d) in LPS-exposed cells. @: cells exposed to LPS treatment significantly decreased compared to KLF1 or KLF2 controls. NS: not significant. Values are means ± SE of at least 4 separate experiments. ^∗∗∗^*P* < 0.001; ^∗∗^*P* < 0.01; ^∗^*P* < 0.05.

As shown above, the full-length *SLC23A2* promoter activity is inhibited by LPS in SH-SY5Y cells (Figure 2(c)). To investigate the molecular basis for this effect in greater depth, we tested whether the LPS inhibitory effect was also apparent on specific regions of the promoter. First, we assessed LPS action on a *SLC23A2* minimal promoter reporter construct (-97 bp to +102 bp; Figure 4(a)) transiently expressed in SH-SY5Y cells. The *SLC23A2* minimal (WT) promoter activity was significantly (*P* < 0.001) inhibited following LPS treatment compared with controls (Figure 4(b)). The minimal promoter region contains one Sp1-binding and two KLF-binding sites. It has been previously established that both transcription factors, Sp1 and KLF, are necessary to drive the basal transcriptional activity of the *SLC23A2* promoter [39, 42]. Therefore, we tested the role of mutations at these sites on the inhibitory LPS effect. Mutant minimal *SLC23A2* promoter constructs were transiently transfected into SH-SY5Y cells. After 24 h of transfection, cells were exposed to LPS for 48 h. Mutational ablation of either KLF-binding site (KLF1 or KLF2) had no effect on the inhibitory action of LPS (Figure 4(b)). In contrast, mutational ablation of the Sp1-binding site led to a loss of the LPS inhibitory effect on the *SLC23A2* promoter activity (Figure 4(b)). Based on this result, we examined the effect on Sp1 protein and mRNA expression in SH-SY5Y cells after exposure to LPS. LPS treatment resulted in significantly (*P* < 0.05 for protein and *P* < 0.001 for mRNA) decreased human Sp1 protein and mRNA levels compared with untreated SH-SY5Y cells (Figures 4(c) and 4(d)). These data suggest that the transcription factor Sp1 mediates the LPS-induced inhibition of neuronal AA uptake.

### 3.5. NF-*κ*B Signaling Regulates the Inhibitory Effect of LPS

**Figure 5:**
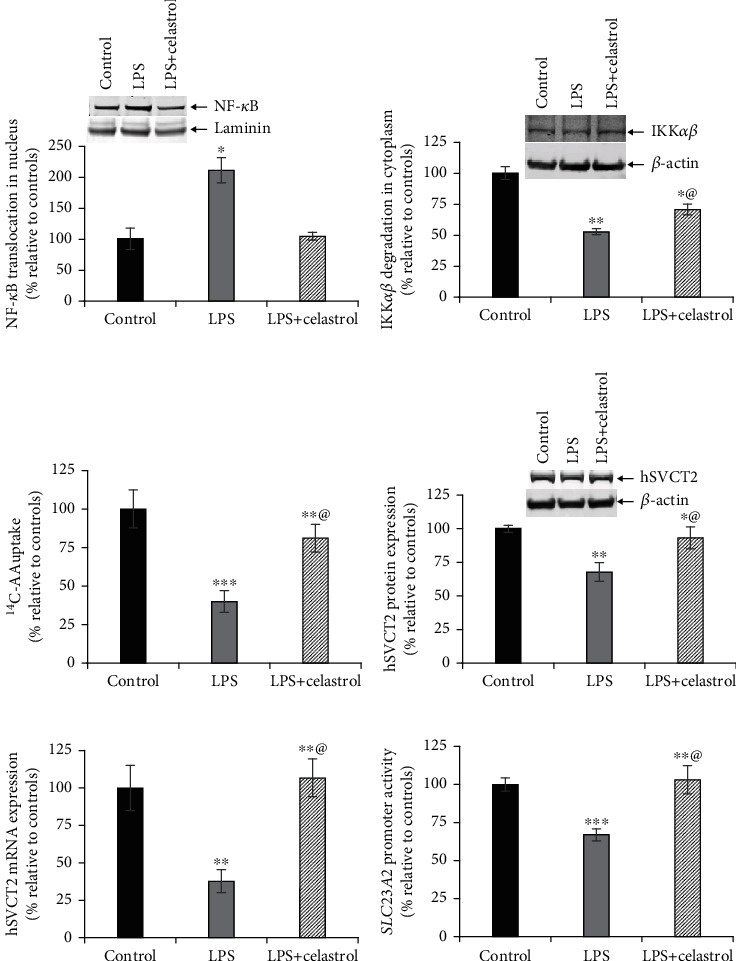
NF-*κ*B signaling pathway plays a role in mediating the LPS-induced inhibitory effect on SVCT2 functional expression. SH-SY5Y cells were pretreated with celastrol (100 nM) for 5 h before LPS treatment; then 48 h later, the NF-*κ*B expression in the nucleus (a), IKK*αβ* expression in the cytoplasm (b), carrier-mediated AA uptake (c), hSVCT2 protein expression (d), hSVCT2 mRNA expression (e), and *SLC23A2* promoter activity (f) were determined. @: cells exposed to LPS plus celastrol treatment significantly recovered versus LPS alone-treated cells. Values are means ± SE of at least 3 separate investigations. ^∗∗∗^*P* < 0.001; ^∗∗^*P* < 0.01; ^∗^*P* < 0.05.

The NF-*κ*B inflammatory signaling pathway is a part of the regulatory mechanism that mediates the action of LPS on gene expression [43–45]. LPS activates the NF-*κ*B pathway in SH-SY5Y cells by driving nuclear translocation of NF-*κ*B and promoting degradation of IKK*αβ* in the cytoplasm (Figures 5(a) and 5(b)). Both these actions were blocked by celastrol, which can act as a NF-*κ*B inhibitor (Figures 5(a) and 5(b)). As Sp1 and NF-*κ*B are often involved in coordinated regulation of gene expression [46–48], we examined whether NF-*κ*B was engaged by LPS to repress hSVCT2 expression. The addition of celastrol to inhibit NF-*κ*B action markedly reversed the effect of LPS-induced inhibition on AA uptake (Figure 5(c)). Celastrol markedly increased the hSVCT2 protein, mRNA expression levels, and *SLC23A2* promoter activity (Figures 5(d)–5(f)). Collectively, these data support the concept that Sp1 and NF-*κ*B signaling pathway coordinate to regulate *SLC23A2* promoter activity in neuronal cells, where NF-*κ*B is activated (Figures 5(a) and 5(b)) and Sp1 is inhibited (Figure 4) by elevated LPS.

## 4. Discussion

The highest concentration of vitamin C is found in the brain, and its levels can be markedly lower in the plasma of patients with neurodegenerative disease [3, 9, 10]. Expression levels of SVCT2 are also markedly lower in human and mouse brain tissue with AD pathology (unpublished observations), which is a possible explanation for this deficit. Recent studies have shown that LPS and other bacterial products are associated with amyloid-beta (A*β*) plaques in AD brains [21, 23], suggesting that abnormal buildup of these bacterial components may be an additional factor to trigger chronic neuroinflammation during the disease course. Brain vitamin C dyshomeostasis induced by inflammation may therefore serve as a mechanism linking inflammation to exacerbated disease phenotypes. LPS and TNF*α* both affect neuronal gene function and expression [30–32, 49, 50]. Here, we investigated their roles in regulating AA uptake and SVCT2 expression in neuronal systems.

Both *in vitro* and *in vivo* assays suggest that the lower levels of SVCT2 functional expression observed upon LPS exposure in neuronal systems are mediated through transcriptional regulation of the *SLC23A2* gene. Substantial evidence shows that Sp1 drives the basal activity of *SLC23A2* promoter in different cellular systems [33, 39, 42] and has also been implicated in transporter regulation in inflammatory conditions [51]. In our investigation, we have used a *SLC23A2* minimal promoter construct expressed in neuronal cells [38, 39] to demonstrate that Sp1 mutation attenuated the LPS-induced decrease in *firefly* luciferase activity (Figure 4). LPS also markedly decreased the Sp1 protein and mRNA expression, signifying that LPS degrades Sp1 and thus reduces *SLC23A2* activity [52].

Sp transcription factors often interact with NF-*κ*B signals mediated at the same DNA binding sites [46–48]. NF-*κ*B is a pleiotropic regulator of many genes responsible for host defense, inflammatory response, and apoptosis [53–56]. The observation that celastrol reversed the inhibitory action of LPS and nuclear translocation of NF-*κ*B implies a convergent regulation of hSVCT2 promoter activity by these dual and often dueling transcription factors. Further work will be needed to resolve the details of how these factors may exert their opposing influences on hSVCT2 promoter activity, possibly even via the very same DNA binding sites. It is worth mentioning that NF-*κ*B is a redox-sensitive transcription factor and regulates SVCT2 mRNA expression in response to redox-state unsteadiness [57], and also, nitric oxide (NO) regulates SVCT2 expression via the NF-*κ*B signaling pathway [58].

In summary, our findings suggest that LPS and TNF*α* downregulate the functional expression of SVCT2, the major vitamin C transporter in the brain. These actions may contribute to the low levels of AA observed during neuroinflammatory insults.

## Figures and Tables

**Table 1:** Oligonucleotide primer combinations used to amplify coding region of the respective genes by RT-qPCR.

Gene name	Forward and reverse primers (5′-3′)
Real-time PCR primers	
hSVCT2	TCTTTGTGCTTGGATTTTCGAT; ACGTTCAACACTTGATCGATTC
hRFVT2	CCCTGGTCCAGACCCTA; ACACCCATGGCCAGGA
hSp1	CCATACCCCTTAACCCCG; GAATTTTCACTAATGTTTCCCACC
h*β*-actin	CATCCTGCGTCTGGACCT; TAATGTCACGCACGATTTCC
mSVCT2	AACGGCAGAGCTGTTGGA; GAAAATCGTCAGCATGGCAA
mNLRP3	ATTACCCGCCCGAGAAAGG; TCGCAGCAAAGATCCACACAG
mTNF*α*	CATCTTCTCAAAATTCGAGTGACAA; TGGGAGTAGACAAGGTACAACCC
m*β*-actin	ATCCTCTTCCTCCCTGGA; TTCATGGATGCCACAGGA
hnRNA primers	
mSVCT2	ACTCTTGTCCATGGCTCTGG; GGGCAAAATCTTCGTTGGGT
m*β*-actin	AGATGACCCAGGTCAGTATC; GAGCAGAAACTGCAAAGAT

## Data Availability

The data that support the findings of this study are available from the corresponding author upon reasonable request.
